# Cording *Mycobacterium tuberculosis* Bacilli Have a Key Role in the Progression towards Active Tuberculosis, Which is Stopped by Previous Immune Response

**DOI:** 10.3390/microorganisms8020228

**Published:** 2020-02-08

**Authors:** Lilibeth Arias, Paula Cardona, Martí Català, Víctor Campo-Pérez, Clara Prats, Cristina Vilaplana, Esther Julián, Pere-Joan Cardona

**Affiliations:** 1 Experimental Tuberculosis Unit (UTE), Fundació Institut d’Investigació en Ciències de la SalutGermans Trias i Pujol (IGTP), 08916 Catalonia, Spain; lilibethariascruz@gmail.com (L.A.); paulacardona29@gmail.com (P.C.); cvilaplana@gmail.com (C.V.); 2 Centro de Investigación Biomédica en Red de Enfermedades Respiratorias (CIBERES), 28029 Madrid, Spain; 3 Centre de Medicina Comparativa i Bioimatge de Catalunya (CMCiB), 08916 Catalonia, Spain; marticatalasabate@gmail.com; 4 Departament de Genètica i de Microbiologia, Facultat de Biociències, Universitat Autònoma de Barcelona, 08193 Catalonia, Spain; Victor.Campo@uab.cat (V.C.-P.); Esther.Julian@uab.cat (E.J.); 5 Bacterial Infections: Antimicrobial Therapies group, Institute for Bioengineering of Catalonia (IBEC), The Barcelona Institute of Science and Technology (BIST), 08028 Catalonia, Spain; 6 Departament de Física, Escola Superior d’Agricultura de Barcelona, Universitat Politècnica de Catalunya—BarcelonaTech, 08860 Catalonia, Spain; clara.prats@upc.edu

**Keywords:** *Mycobacterium tuberculosis*, cording, C3HeB/FeJ mice, neutrophils, BCG, CXCL-1, neutrophilic extracellular traps

## Abstract

Cording was the first virulence factor identified in *Mycobacterium tuberculosis* (Mtb). We aimed to ascertain its role in the induction of active tuberculosis (TB) in the mouse strain C3HeB/FeJ by testing the immunopathogenic capacity of the H37Rv strain. We have obtained two batches of the same strain by stopping their growth in Proskauer Beck liquid medium once the mid-log phase was reached, in the noncording Mtb (NCMtb) batch, and two days later in the cording Mtb (CMtb) batch, when cording could be detected by microscopic analysis. Mice were challenged with each batch intravenously and followed-up for 24 days. CMtb caused a significant increase in the bacillary load at an early stage post-challenge (day 17), when a granulomatous response started, generating exudative lesions characterized by neutrophilic infiltration, which promoted extracellular bacillary growth together with cording formation, as shown for the first time in vivo. In contrast, NCMtb experienced slight or no bacillary growth and lesions could barely be detected. Previous Bacillus Calmette-Guérin (BCG) vaccination or low dose aerosol (LDA) Mtb infection were able to delay the progression towards active TB after CMtb challenge. While BCG vaccination also reduced bacillary load when NCMtb was challenged, LDA did not, and its proliferative lesions experienced neutrophil infiltration. Analysis of lung cytokine and chemokine profiles points to their capacity to block the production of CXCL-1 and further amplification of IL-1β, IL-17 and neutrophilic extracellular trap formation, all of which are essential for TB progression. These data highlight the key role of cording formation in the induction of active TB.

## 1. Introduction

Tuberculosis (TB) is still a major threat for humankind, causing 10 million new cases and 1.5 million deaths every year [1]. *Mycobacterium tuberculosis* (Mtb) infection is caused by infected aerosols generated by patients with TB, which enter the alveolar space and are engulfed by alveolar macrophages [2]. However, the majority of subjects infected do not generate symptomatology or lung damage; in fact, it has been calculated that 25% of humankind is already infected [3], and it is generally accepted that around 10% of these people will generate TB [4]. Progression from infection to disease is a multifactorial process determined by environmental aspects, such as dose exposure or air pollution; host susceptibility, such as genetic propensity, comorbidities or even the localization of the infection (i.e., upper lobes are more susceptible); and also by bacterial virulence factors [5,6].

From the very first moment of identifying Mtb as the etiological cause of TB, Robert Koch described the bacilli, which are usually found as small groups arranged in bundles, suggesting the cording morphology [7]. It was not until several decades later, that Middlebrook et al. identified a positive correlation between virulence in Mtb strains and cording formation [8]. Recently the role of cording has been questioned, since ultrastructural studies with scanning electron microscopy (SEM) have demonstrated that nonpathogenic mycobacteria also produce cording and that cording producing strains are also pathogenic for the macrophages, as in the case of *Mycobacterium abscessus* [9,10]. In this work, Julian et al. [10] highlighted the role of bacilli aggregation in which the orientation of the long axis of each cell was parallel to the long axis (cording) or were not oriented and merely forming irregular clumps.

Recently, Kalsum et al. have demonstrated that cording induces the formation of macrophage extracellular traps (METs) in human monocyte-derived macrophages, a process linked with the secretion of early secreted antigenic target-6 (ESAT-6) [11]. Furthermore, Mahamed et al. have been able to demonstrate that, in primary human macrophages, Mtb aggregates cause macrophage death and that phagocytosis of large aggregates induces more cytotoxicity than small aggregates containing similar numbers of bacilli [12]. In this study, the authors showed that Mtb doubling time was reduced when growing inside dead macrophages compared with extracellular growth (24.7 vs. 36.1 h). All this information can be linked with the immunopathology found in the model of active TB in the C3HeB/FeJ mice. Contrary to what happens with the majority of other mouse strains [13], Mtb challenge in this strain is able to generate the whole spectrum of lesions seen in humans [14], including liquefied lesions, which are essential to induce cavitation [5]. This is because this mouse generates a neutrophilic infiltration of the lung, which resembles the progression towards active TB [6,15]. In this process, neutrophilic extracellular traps (NETs) are generated, which fuel the extracellular growth of Mtb [16]. This is why this mouse strain provides the most accurate evaluations of virulence factors related to TB induction.

Taking advantage of our previous knowledge of the dynamics of cording formation in liquid culture [17], we have generated two batches of the same strain of Mtb, one with and one without cording, to ascertain the role of previous cording formation in the progression towards TB. Our data demonstrate the higher capacity of cording bacilli to induce active TB, due to the induction of a pulmonary proinflammatory response, and how this can be stopped by previous BCG vaccination or low dose aerosol (LDA) infection.

## 2. Materials and Methods

### 2.1. Batch Production and Experimental Design

Five colonies from a 4-week culture of Mtb H37Rv Pasteur strain in Middlebrook 7H11 agar plates (BD Diagnostics, Franklin Lakes, NJ, USA) were grown in Proskauer Beck medium containing 0.01% Tween 80 at 37 °C in a shaking incubator to the mid-log phase (MP), OD (Abs 595 nm) 0.5 in the case of the noncording Mtb (NCMtb) batch or MP+2 days in the cording Mtb (CMtb) batch and kept in vials at −80 °C until used.

Three experiments were conducted to evaluate differences in TB progression due to previous cording formation. Animals were infected with the same CFU of NCMtb and CMtb of Mtb H37Rv Pasteur strain. Inoculum dose was assessed after infection after plating serial dilutions in Middlebrook 7H11 agar plates. Two different protection strategies, BCG vaccination and LDA infection, were also evaluated in both batches.

In experiment 1, animals were intravenously (IV) infected with 4 × 10^4^ colony-forming units (CFU) of the CMtb and NCMtb. At days 17, 21 and 24 post-infection (PI), animals were euthanized (*n* = 3 per group and time-point; except for CMtb d24 PI, *n* = 4). At each time-point, bacillary load (BL) and pathology were evaluated in lung samples. At day 24, lung samples were obtained to assess cording formation in vivo by SEM.

In experiment 2, animals were subcutaneously vaccinated with 10^6^ CFU of BCG Live U.S.P vaccine (SII-ONCO-BCG^®^). After 6 weeks, animals were IV infected with 4 × 10^4^ CFU of CMtb and NCMtb. At days 17, 21 and 24 PI, animals were euthanized (*n* = 4 per group at day 17 PI and *n* = 5 per group at days 21 and 24 PI). At each time-point BL, pathology and immune response were evaluated in lung samples.

In experiment 3, animals were challenged with LDA infection to deliver around 50 CFU of NCMtb to the lungs using an airborne infection apparatus (Glas-col Inc., Terre Haute, IN, USA). After 6 weeks, animals were IV infected with 4 × 10^4^ CFU of CMtb and NCMtb. At days 17, 21 and 24 PI, animals were euthanized (*n* = 5 per group and time-point). At each time-point, BL, pathology and immune response were evaluated in lung samples.

### 2.2. Image Analysis of Bacillary Aggregates

Ten 20 µL drops from NCMtb and CMtb were fixed on a glass slide and stained using the Ziehl–Neelsen procedure. Three pictures per drop were taken using an Eclipse 50i microscope (Nikon, Tokyo, Japan) equipped with a DS-Fi 1 camera (Nikon, Tokyo, Japan) at 100× using the NISElements D version 3.0× software package (Nikon Instruments Inc., Tokyo, Japan). Single cells and aggregates were detected by image analysis using the MATLAB software (MATLAB, vs. 9.3; The MathWorks^®^).

The original colored image was first converted into a grey-scale image, and then a threshold was fixed to 5% convert the image into a black and white one. The black isolated regions were defined as “spots”, which could be any particle detected (from single bacilli to large cords). Areas under 1 µm^2^ were not considered. Area was determined for each aggregate in all pictures taken, 30 for each batch. Distribution of areas was calculated as the frequency of spots in eight area intervals, logarithmically distributed between 1 and 10^4^ μm^2^.

### 2.3. Animals and Ethics

All procedures were performed according to protocols DMAH9091 and DMAH9071, which were reviewed by the Animal Experimentation Ethics Committee of the Hospital Universitari Germans Trias i Pujol (registered as B9900005) and approved by the Departament d’Agricultura, Ramaderia, Pesca, Alimentació i Medi Natural of the Catalan Regional Government, according to current national and European Union legislation regarding the protection of experimental animals. Mice were supervised daily following a strict monitoring protocol in order to ensure animal welfare and euthanized, if required, with isoflurane (inhalation excess).

Male and female C3HeB/FeJ specific-pathogen-free mice (5–8 and 14–15 weeks old) were bred in the barrier facility of the Centre for Comparative Medicine and Bioimage of Catalonia (CMCiB), and all procedures were conducted in the CMCiB-BSL-3 facility. Animals were maintained on a 12 h light–dark cycle in a temperature- and humidity-controlled room.

### 2.4. BL

Left lung samples from each animal were collected and homogenized, and several dilutions plated on nutrient Middlebrook 7H11 agar. Visible CFU were counted after incubation for 28 days at 37 °C.

### 2.5. Pathology

Right lower lung lobe samples were fixed in 10% buffered formalin, embedded in paraffin and 5-µm sections stained with hematoxylin-eosin for microscopic observation and analysis of the damaged area using the NISElements D version 3.0× software package (Nikon Instruments Inc., Tokyo, Japan). Four recuts of a block containing all group samples were used to determine the damaged area as a percentage of total lung area. In the first experiment, each animal was analyzed separately.

### 2.6. Ex Vivo SEM

To fix mice lungs and prevent their collapse, the trachea was cut and a fixative solution based on 2.5% (*v/v*) glutaraldehyde and 2% (*w/v*) paraformaldehyde (EM grade, Merck, Darmstadt, Germany) in phosphate buffer (PB; 0.1 M, pH 7.4; Sigma-Aldrich, Steinheim, Germany) was perfused, using a syringe attached to a catheter, until the entire bronchial tree was filled. This step is important to preserve the internal pulmonary structure as well as to ensure the fixation of all alveolar spaces.

Lungs were kept in fixative solution for approximately 12 h. Afterwards, lungs were post-fixed for 2 h with 1% (*w/v*) osmium tetroxide (TAAB Lab., Aldermaston, UK), followed by washes with deionized water and sequential dehydration by ascending ethanol series (50%, 70%, 80%, 90% and 95% for 10 min each and twice with 100% ethanol).

For SEM analysis, the fixed lungs were cut with a blade at different heights and directions, placed on stubs and metallized (Emitech K550X Sputter Coater Metallizer) using a gold-palladium alloy. Finally, samples were observed using Zeiss EVO^®^ MA 10 scanning microscope (Zeiss, Oberkochen, Germany) operating at 15 kV in the Electronic Microscopy Service at the Universitat Autònoma de Barcelona.

### 2.7. Immune Response

A cytokine profile study was performed in lung homogenates from right upper and middle lobes. The following cytokines were measured by Luminex xMAP^®^ technology: IFN-γ, TNF-α, IL-1β, IL-6, IL-17, IL-10, TGF-β, CXCL1 and CXCL5. Results are expressed as pg per mL of supernatant. The assay was performed with the MILLIPLEX^®^ MAP kit (EMD Millipore Corporation, Billerica, MA, USA), following the manufacturer’s instructions, and analyzed with xPONENT Software (Luminex Corporation, Austin, TX, USA).

### 2.8. Statistics

The nonparametric Mann–Whitney test was used to compare between groups in BL, pathology and immune response, using GraphPad Prism (GraphPad software v7.0, La Jolla, CA, USA).

The immune response in lungs was also analyzed by principal component analysis (PCA), using RStudio (version 1.1.463). PC1 and PC2 represent the two principal components that contribute to most of the variance among samples. The direction of the arrows and their lengths represent the contribution and strength of every variable to each principal component. Cytokine concentration levels were scaled to unit variance. PC1 differences were analyzed using one-way ANOVA and Sidak’s multiple comparison, using GraphPad Prism (GraphPad software v7.0, La Jolla, CA, USA).

Statistically significant differences are designated as follows: * *p* < 0.05; ** *p* < 0.01; *** *p* < 0.001.

## 3. Results

### 3.1. Cording Mtb Induces a Significant Change in the Progression of the Infection

**Figure 1:**
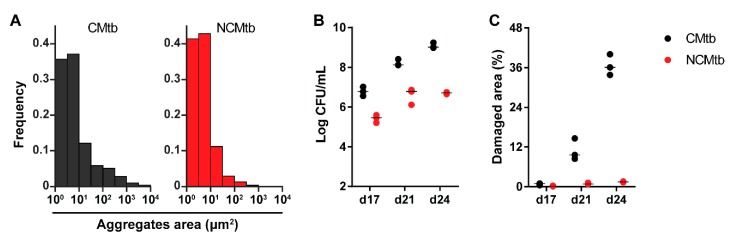
Impact of cording bacilli challenge on the progression towards active tuberculosis (TB). (**A**) Aggregate area distribution pattern for both batches. Each column represents the frequency of spots for each area interval, logarithmically distributed between 1 and 10^4^ μm^2^. (**B**) Bacillary load (BL) and (**C**) damaged lung area progression after intravenous (IV) challenge with cording Mtb(CMtb) or non-cording Mtb(NCMtb).

Figure 1A shows the areas of the particles measured, under ×100 amplification, demonstrating cording formation in the CMtb compared to NCMtb [17]. Despite challenging with the same CFU, CMtb increased BL and damaged area in lung when compared with NCMtb (Figure 1B,C). It is noteworthy that after a slight increase, by day 21 the progression of NCMtb reached a plateau of BL, causing almost no damage to lungs. CMtb caused mainly exudative lesions from day 17, and at day 21, large necrotic lesions were detected. In the case of NCMtb, proliferative and incipient exudative lesions of smaller sizes were seen even when the latter were not detected until day 21 (Figure S1).

### 3.2. Cording Bacilli Can Be Detected in Exudative Lesions

**Figure 2:**
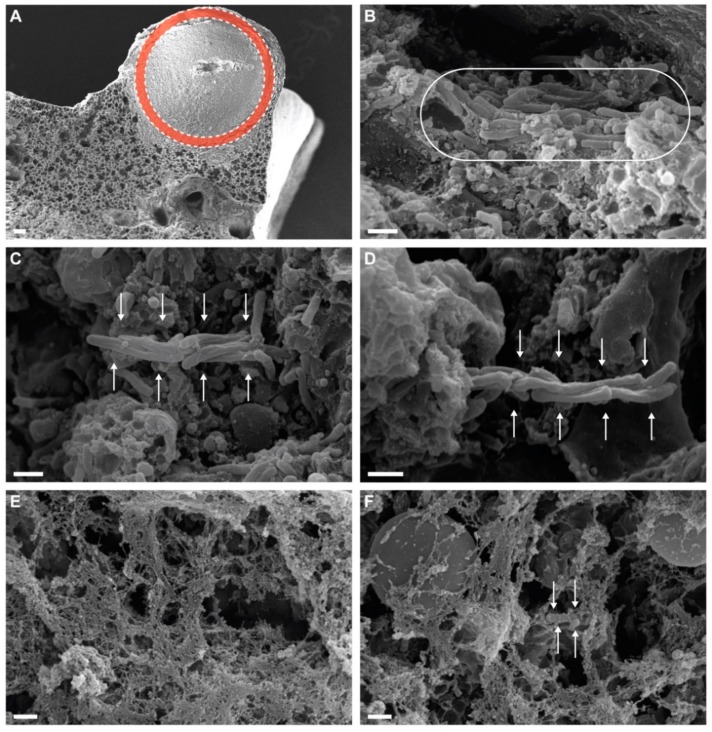
Induction of cording in vivo. SEM micrographs of exudative lesions showing in vivo cording formation. Lower amplification showing one of the representative lesions found in lungs from CMtb mouse. (**A**) One of the zones explored in depth in the lungs is marked with a red circle. Formation of cording bacilli is marked with a white ellipse (**B**) or white arrows (**C**,**D**). (**E**) The formation neutrophilic extracellular traps (NETs). (**F**) Detail with trapped bacilli. Scale bars equal 100 μm in (**A**) and 1 μm in the others.

Analysis of large exudative lesions with SEM at day 24 PI with CMtb showed the formation of cording in vivo, characterized by the orientation of each cell in parallel to the long axis in the context of NET formation (Figure 2).

### 3.3. BCG Vaccination Protects Against TB Reducing the Inflammatory Response

**Figure 3:**
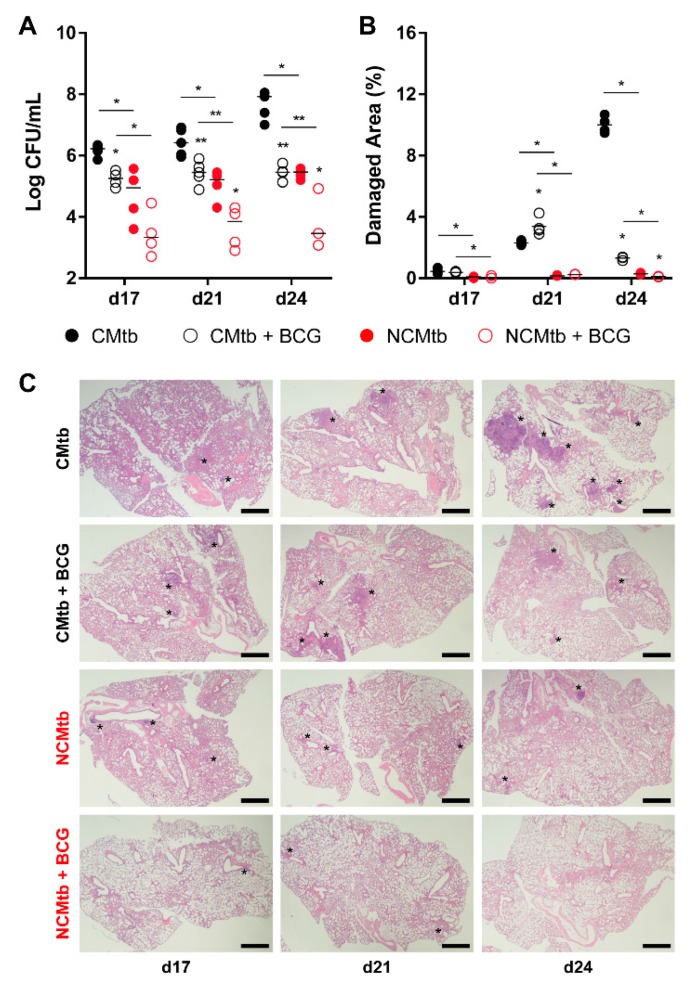
Protective effect of BCG immunization. (**A**) Progression of pulmonary BL, (**B**) lung damaged area and (**C**) evolution of the lesions. Each circle represents an animal (**A**) or a recut (**B**) and lines are medians. Asterisks indicate differences within the same batch; bar and asterisks indicate differences between batches. Mann–Whitney test (* *p* < 0.05, ** *p* < 0.01). Scale bars equal 1 mm and asterisks point lung lesions.

BCG vaccination had a significant impact in the reduction of the BL in both CMtb and NCMtb groups, stopping the bacillary growth (Figure 3A). This was also seen when analyzing the damaged area (Figure 3B). Looking at the quality of the granulomas, CMtb caused higher neutrophil infiltration, which translated to larger exudative lesions with necrotic areas at day 24. This effect was dramatically reduced after BCG vaccination, where an important lymphocytic infiltration could be detected replacing the neutrophilic one (Figure 3C and Figure S1).

**Figure 4:**
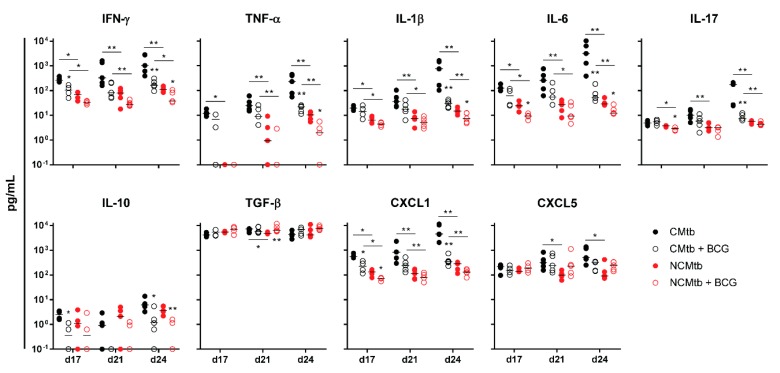
Impact of BCG vaccination on the immune response in lungs. Analysis of inflammatory mediators in lung homogenates at days 17, 21 and 24 PI. Results are represented as log10 of the concentration in pg/mL. Each circle represents an animal and lines are medians. Asterisks indicate differences within the same batch; bar and asterisks indicate differences between batches. Mann–Whitney test (* *p* < 0.05, ** *p* < 0.01).

IFN-γ and CXCL-5 levels did not show major changes over time in any experimental group (Figure 4). On the contrary, IL-1β, IL-6, IL-17 and CXCL-1 levels exhibited a progressive increase in the non-vaccinated CMtb group, while keeping relatively constant in the others. Overall, BCG vaccination reduced the levels of all cytokines and chemokines except TGF-β and CXCL-5. Of all of them, it is interesting to note that on day 17, BCG vaccination significantly reduced CXCL-1 levels in both CMtb and NCMtb. At this time-point, vaccination also reduced the levels of IFN-γ and IL-10 in CMtb and IL-6 and IL-17 levels in the case of NCMtb. TNF-α levels also experimented an increase in NCMtb, which was delayed after BCG vaccination (Figure 4). Thus, vaccination with BCG was able to stop the progression of Mtb in both cording and non-cording bacilli by significantly modulating the immune response and therefore the damaged area (Figure 3).

Regarding the PCA analysis, the first component (PC1) explained between 63.8% and 79.2% of the variation among samples (Figure S2), depending on the time-point. Overall, the main contributions came from CXCL-1, IL-1β, IL-6, TNF-α, IL-17 and IFN-γ. Looking at the PC1 index, there is a significant reduction after vaccination in CMtb but no changes in the case of NCMtb, suggesting that inflammation is not high enough in the latter to be influenced by BCG.

### 3.4. LDA Protects Against TB Progression after CMtb Challenge

LDA was allowed to progress for 6 weeks, generating proliferative lesions, defined by the presence of macrophages, lymphocytes, scanty neutrophils and foamy macrophages at the periphery (Figures S1 and S3), as previously described [18]. Impact of IV challenge could be detected by day 17 with the presence small granulomas based mainly by scarves of lymphocytes in the case of NCMtb and a mixture of lymphocytes plus neutrophils, in CMtb. Both batches caused neutrophil infiltration in LDA proliferative lesions (Figure S1).

**Figure 5:**
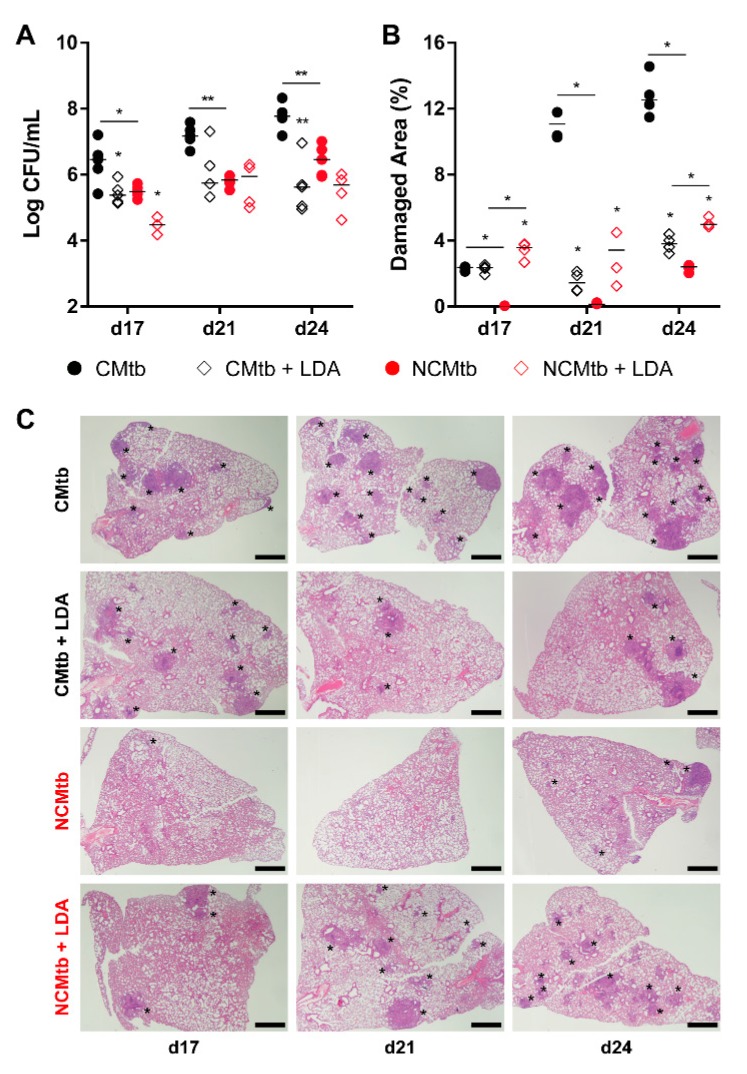
Protective effect of low dose aerosol (LDA) infection. (**A**) Progression of pulmonary BL, (**B**) lung damaged area (**C**) and evolution of the lesions. Each circle represents an animal (**A**) or a recut (**B**) and lines are medians. Asterisks indicate differences within the same batch; bar and asterisks indicate differences between batches. Mann–Whitney test (* *p* < 0.05, ** *p* < 0.01). Scale bars equal 1 mm and asterisks point lung lesions.

In the case of CMtb infection, BL reduction after LDA was equivalent to BCG vaccination (Figure 5), also reducing the neutrophil infiltration and increasing the lymphocytic one (Figure S1). LDA infection only reduced significantly the BL of NCMtb at day 17 PI but increased the damaged area (Figure 5).

**Figure 6:**
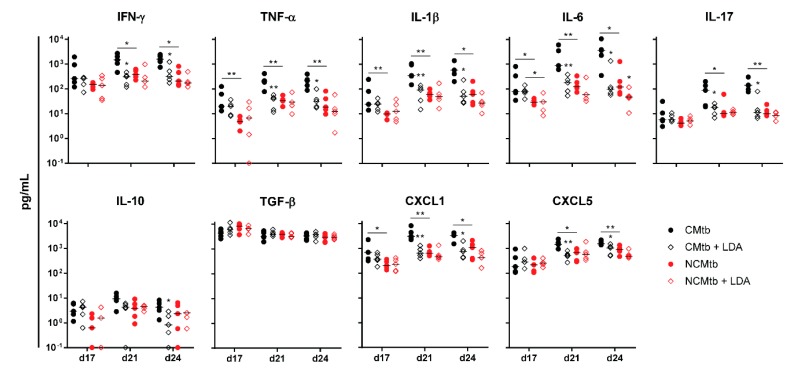
Impact of LDA infection in the immune response in lungs. Analysis of inflammatory mediators in lung homogenates at days 17, 21 and 24 PI. Results are represented as log10 of the concentration in pg/mL. Each circle represents an animal and lines are medians. Asterisks indicate differences within the same batch; bar and asterisks indicate differences between batches. Mann-Whitney test (* *p* < 0.05, ** *p* < 0.01).

In terms of the immune response, this can also be correlated with the presence of high levels of IFN-γ at the beginning of the infection in all experimental groups (i.e., >10^2^ pg/mL), unlike the case of BCG vaccination (Figure 6). This uniform response was not reflected in the BL, showing different values led by the group that was only challenged with the CMtb. Contrary to what happened with BCG vaccination, the kinetics of the inflammatory response appear to follow the same shape in all groups, reaching a plateau at day 21. Of note, at day 17 BL appears to be slightly higher than in the previous experiment, so the infection is at a much more mature stage. Interestingly, at day 17 CXCL-1 was also reduced by LDA infection in the CMtb, although not significantly. In the case of NCMtb, LDA did not change the levels of cytokines and chemokines.

**Figure 7:**
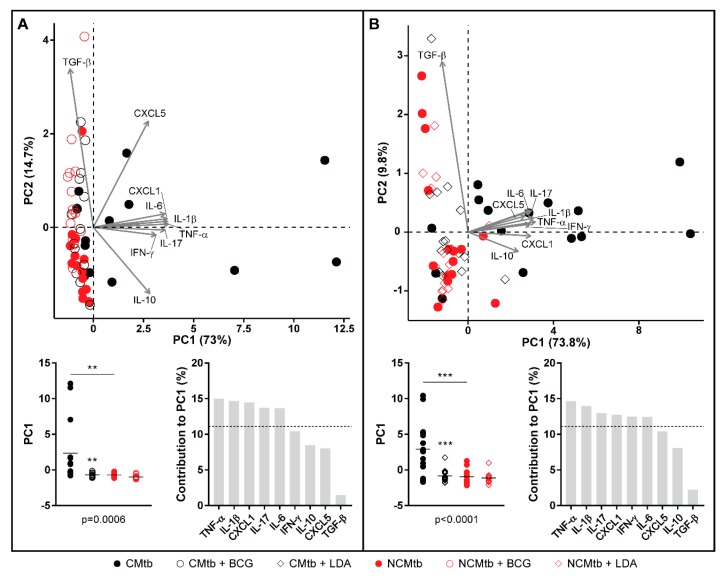
Comparison of PCA of inflammatory mediators after BCG vaccination (**A**) or LDA infection (**B**) in lung homogenates. PCA biplot showing each animal and variable (top). PC1 scores for each animal, lines are means; ANOVA and Sidak’s multiple comparisons test (** *p* < 0.01, *** *p* < 0.001) (bottom left). Mediators’ contribution to PC1 (bottom right).

Concerning the PCA analysis, the first component (PC1) explained between 68.2% and 79.8% of the variation among samples (Figure S4), depending on the time-point. There is a significant reduction in the PC1 index after LDA in CMtb. Once again, the main contributions came from CXCL-1, IL-1β, IL-6, TNF-α, IL-17 and IFN-γ, with a similar trend to that observed after BCG vaccination (Figure 7). Both protection strategies analyzed reduced the proinflammatory PC1 index in CMtb.

## 4. Discussion

The experimental C3HeB/FeJ TB model has been very important in studies to explain how Mtb infection progresses to active TB. As has been previously described, the main difference between a lesion in a latently infected person and one from a person with active TB is its size [19]. To reach this greater size, a very intense inflammatory response must be triggered so as to be able to surpass the encapsulation process that takes place in the lungs of large mammals [20]. In the C3HeB/FeJ model, some Mtb lesions develop in a way that resembles human ones, as exudative large intragranulomatous necrosis that finally liquify [16]. The process requires a rapid enlargement of the lesions, thanks to a massive neutrophilic infiltration and NETs that allow fast extracellular bacillary growth, the formation of new “daughter” lesions in the vicinity and a final coalescence of the lesions [21]. Our data show how this infiltration is able to favor the induction of cording bacilli and thus perpetuates the exudative response by causing the necrosis of macrophages [12] and triggering the attraction of neutrophils [22,23].

Recent data from Wood et al. on the quality of infective aerosols generated by TB patients show that around 1% of them can carry cording bacilli [24]. These bacilli have a good chance of developing an exudative lesion and one that is able to progress rapidly enough to avoid the local encapsulation process that can stop it from forming at all [20]. This could explain the higher probability of TB progressing during the first year after infection [25,26], especially in the pediatric population. We can speculate that the “chance” then depends on the capacity of cording bacilli formed in situ to disseminate or to generate a “daughter lesion”, thus increasing the possibility of generating another exudative lesion that is able to further progress [21]. In the adult immunocompetent population this has to be accompanied by the localization of the lesion in the upper lobe, where the process of bacillary accumulation benefits from the low capacity for bacillary drainage due to mechanical constraints [6,27]. In this regard, our data support the relevance of cording in Mtb bacilli at the time of infection, by locally concentrating a higher bacillary load that is able to bias the immune response towards a proinflammatory state, which attracts neutrophils to the granuloma and generates an exudative lesion, which in turn increases in size, necrotizes and rapidly progresses towards active TB.

We wanted to assess the role of a previous immune response triggered both by either BCG vaccination or LDA in this system. BCG vaccine is the only vaccine available against TB, and so far there is a consensus on using it in neonates to avoid the progression towards mortal clinical forms of TB at this age, i.e., meningitis and miliary, but there is no consensus on its value for protection against infection or pulmonary TB, especially in adults [28]. Previous infection has also been linked to protection, as was demonstrated in the field by Heimbeck and by the observation of protection against active TB in student nurses who were already infected [29], recently highlighted by Bloom [30]. In our work we wanted to elucidate the impact of these two interventions at the time when the granulomatous response is triggered in this system [16].

Our data show the relevance of previous immunization to the control of BL and lung damage after reinfection. However, challenging with CMtb reveals that there are some limitations. It appears that there is a nonresponding bacillary population, which may correspond to the one that is growing extracellularly in the necrotic lesions and thus is not being controlled by the protective effect of macrophages activated by IFN-γ. It can be speculated that at this point the bacillary population immersed in the NETs can barely be managed by activated macrophages, and thus the Th1 response is less effective, which allows the process of neutrophil infiltration and perpetuates the extracellular growth of the bacilli and thus the expansion of the lesion. This brings us to the fact that a good protective response has to deal with the exacerbated inflammatory response, as has been highlighted previously [31]. All this provides more support to the need for host-oriented therapies in order to control TB [32].

Additionally, our data suggest that reduction of CXCL-1 levels could be the clue. Recently, Boro et al. [33] have shown how important it is to block the CXCL-1 system to stop the amplification of the inflammasome, and thus the production of IL-1β, in the context of Mtb infection, together with IL-17 production, ROS production and NET formation. Lombard et al. [34] also identified CXCL-1, but included CXCL-5, as the major recruiters of neutrophils. In our work, CXCL-5 seems to make little contribution to the initial inflammatory process, in a similar way to the anti-inflammatory (TGF-β and IL-10) process, although with time it integrates into the inflammatory component. Lombard also highlights that the IL-17A-driven exacerbation of inflammation observed in several circumstances may lead to tissue destruction. IL-17A is overproduced in response to high mycobacterial loads in the mouse [35] or in humans infected with multidrug-resistant Mtb strains resulting in high antigen loads [36], leading to an unfavorable clinical outcome.

We have also observed how IV challenge increases the neutrophil infiltration and the damage response in previous proliferative lesions. This could correspond to the effect seen in the zebrafish by Davis and Ramakrishna, where reinfecting bacilli were attracted to primary lesions [37], and also highlights the mechanism of systemic dissemination of the bacilli after aerosol infection as a potential mechanism of progression. It also supports the mechanism of TB progression defended by classical TB specialists like Pottenger or Canetti [6,38,39]. These authors defended the idea that the more often you are reinfected, the higher the chances of developing active TB are, especially if the reinfection is in an already active lesion. This could be a mechanism for progression towards TB, especially in high incidence countries. Certainly, in this regard, the experience of our group with multiple consecutive aerosol infections supports this view [18].

Although the infection route used in these experiments does not resemble the real one, the results obtained have shown the importance of cording, which should be therefore explored in further experiments using aerosol as infection route. We have used the IV route because is the one that is able to generate exudative “human-like” lesions with liquefaction in a predictable timeframe, giving the chance to determine which mechanisms precede this generation. Aerosol challenge of C3HeB/FeJ mice can induce these lesions, but in a wider unpredictable timing [14,40,41] to the point that some mice cannot develop them even at week 26 after challenge [42]. C3HeB/FeJ reacts differently than other laboratory mouse strains. IV challenge is less pathogenic in C57BL/6 or BALB/c than low dose aerosol, even after IV inoculation of a higher bacillary load (up to 10^5^ CFUs). This particularity was firstly described by North [43]. The explanation is in the faster infection of the spleen, which raises a quicker protective immune response [44] even in mice infected concurrently with both routes [45]. Our data also highlight the importance of assessing the quality of the Mtb challenge and the need to check for cording in any future challenge experiments, either ex vivo or in vivo, as this factor has an enormous relevance at the moment of assessing the nature of the immune response that is being evaluated.

## 5. Conclusions

Overall, our data highlight the fact that the presence of cording bacilli in the infecting dose is the hallmark for increased probability that an Mtb infection will progress towards active TB in naïve hosts.

## Supplementary Materials

Supplementary materials can be found at https://www.mdpi.com/2076-2607/8/2/228/s1. Figure S1: Microscopic characteristics of the lesions. Figure S2: PCA analysis and evolution through time after BCG vaccination. Figure S3: PCA analysis and evolution through time after low dose aerosol infection. Figure S4: Impact of LDA infection in the immune response in lungs.
