# Supplementary material for: Cording Mycobacterium tuberculosis Bacilli Have a Key Role in the Progression towards Active Tuberculosis, Which is Stopped by Previous Immune Response
Source: Microorganisms. 2020 Feb 8;8(2):228. doi: 10.3390/microorganisms8020228 (PMC7074780; doi:10.3390/microorganisms8020228)
Supplement: Supplementary file 1 [file microorganisms-08-00228-s001.pdf]

microorganisms

CMtb		NCMtb		CMtb + BCG		NCMtb + BCG		CMtb + LDA				NCMtb + LDA			
								Aer Lesions		IV lesions		Aer Lesions		IV lesions	
Panel	day PI	Panel	day PI	Panel	day PI	Panel	day PI	Panel	day PI	Panel	day PI	Panel	day PI	Panel	day PI
A	17	G	24	K	17	O	17	R	17	U	17	Y	21	Z	24
B	21	H	21	L	17	P	24	S	17	V	17			AA	24
C	17	I	21	M	21	Q	21	T	21	W	21			AB	17
D	21	J	24	N	21					X	21			AC	17
E	21													AD	17
F	24														

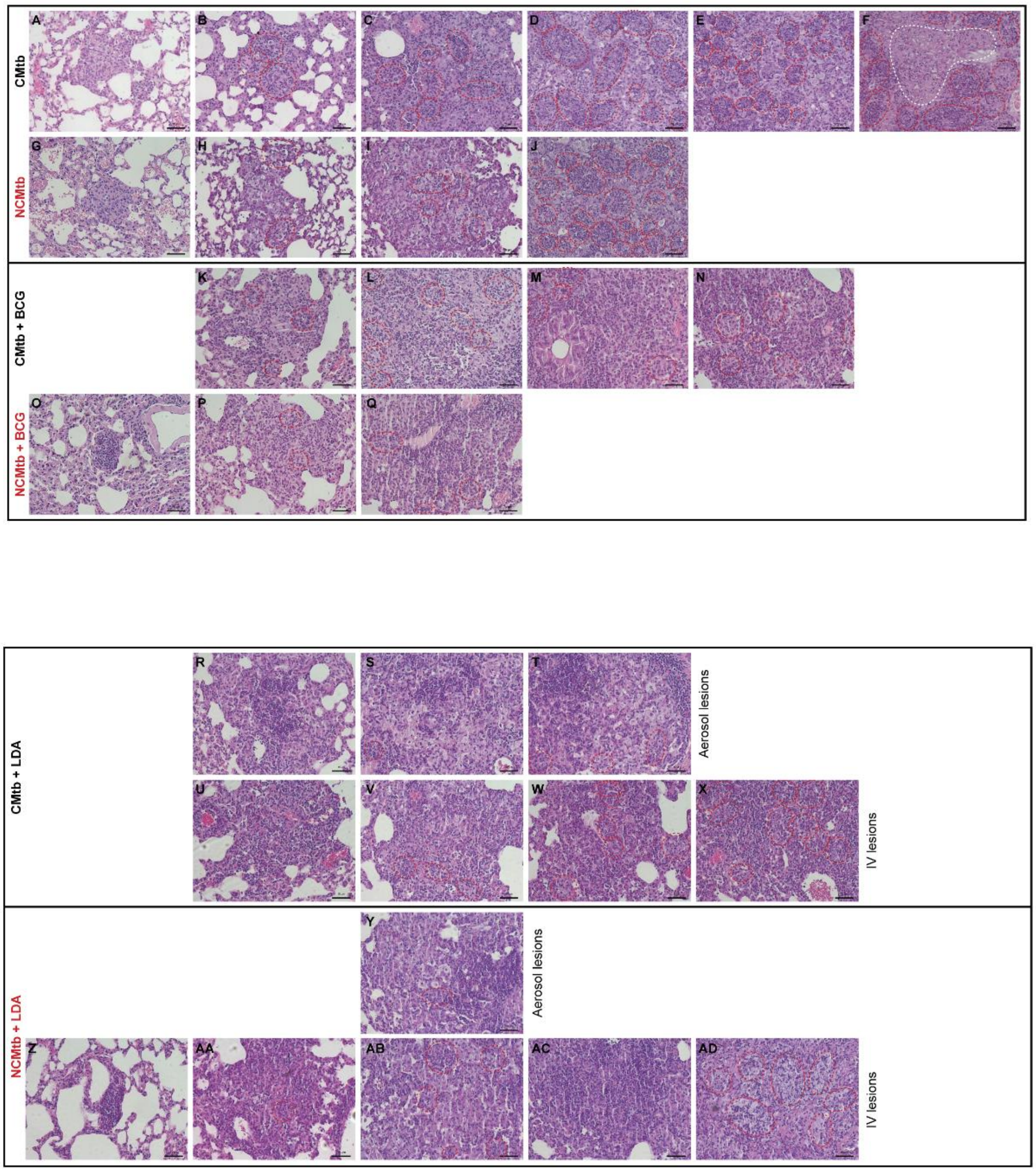

Figure S1. Microscopic characteristic of the lesions. Different types of lung lesions for each experimental group, evolving from left to right. Red dotted circles mark neutrophil infiltration and white marks central necrosis of the lesion. HE staining, 200x magnification, scale lines represent 50 µm. The day post-infection from each picture is detailed in the following table.

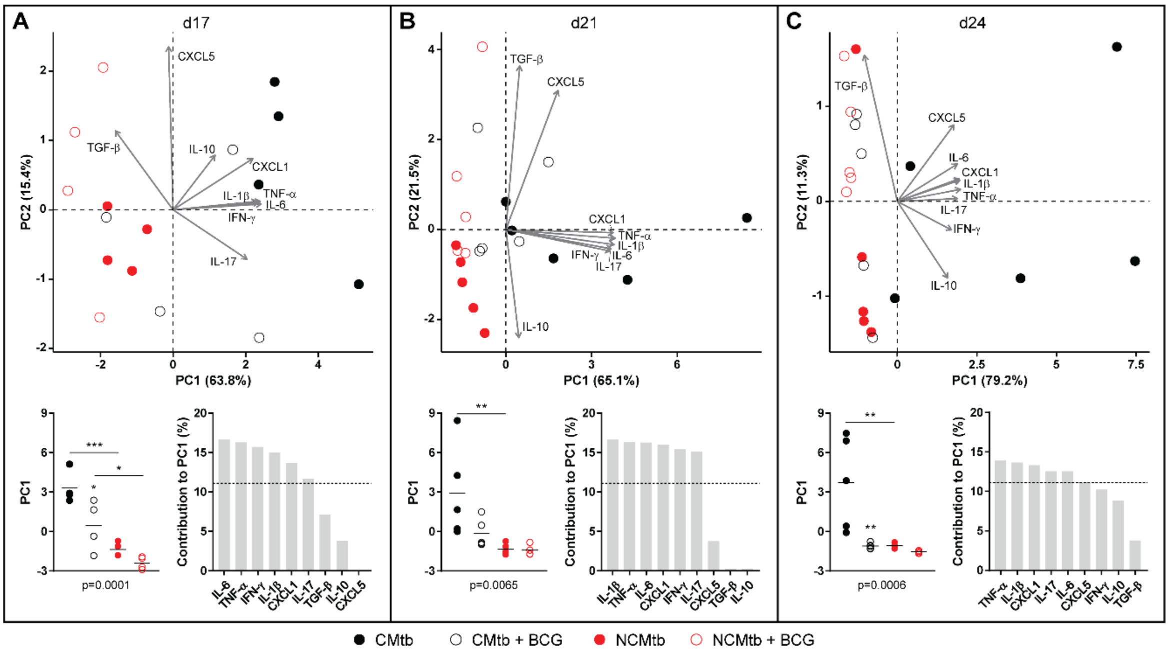

Figure S2. Impact of BCG vaccination in the immune response in lungs. PCA of immune mediators in lungs homogenates at days 17 (**A**), 21 (**B**) and 24 (**C**) p.i. PCA biplot showing each animal and variable (top). PC1 scores for each animal, lines are means; asterisks indicate differences within the same batch; bar and asterisks indicate differences between batches; ANOVA and Sidak's multiple comparisons test (*$p < 0.05$, **$p < 0.01$, ***$p < 0.001$) (bottom left). Mediators contribution to PC1 (bottom right).

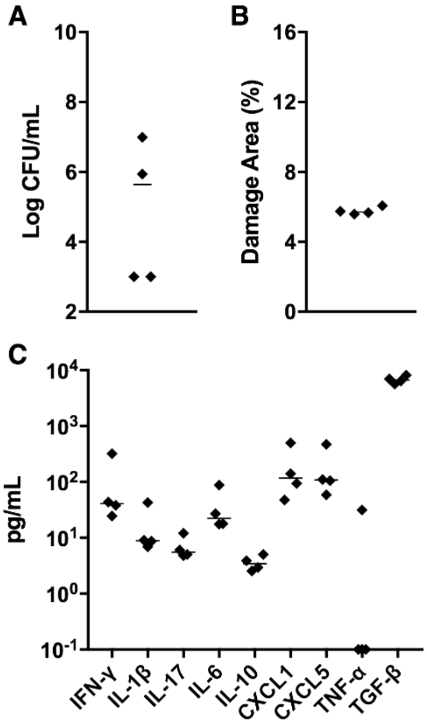

Figure S3. Evolution of LDA infection. (**A**) Bacillary load, (**B**) damaged lung area and (**C**) inflammatory mediators at week 6 post-LDA infection and prior to CMtb and NCMtb IV infection.

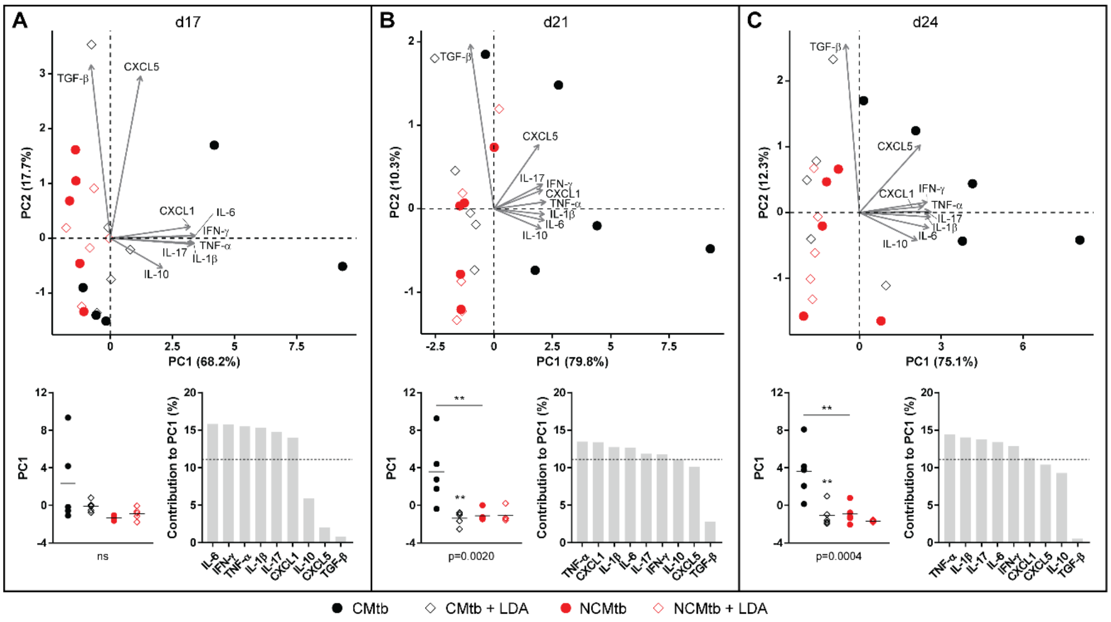

Figure S4. Impact of LDA infection in the immune response in lungs. PCA of immune mediators in lungs homogenates at days 17 (**A**), 21 (B) and 24 (**C**) p.i. PCA biplot showing each animal and variable (top). PC1 scores for each animal, lines are means; asterisks indicate differences within the same batch; bar and asterisks indicate differences between batches; ANOVA and Sidak's multiple comparisons test (*$p < 0.05$, **$p < 0.01$, ***$p < 0.001$) (bottom left). Mediators contribution to PC1 (bottom right).
